# Comparative genomics of *Stutzerimonas balearica* (*Pseudomonas balearica*): diversity, habitats, and biodegradation of aromatic compounds

**DOI:** 10.3389/fmicb.2023.1159176

**Published:** 2023-05-15

**Authors:** Francisco Salvà-Serra, Danilo Pérez-Pantoja, Raúl A. Donoso, Daniel Jaén-Luchoro, Víctor Fernández-Juárez, Hedvig Engström-Jakobsson, Edward R. B. Moore, Jorge Lalucat, Antoni Bennasar-Figueras

**Affiliations:** ^1^Microbiology, Department of Biology, University of the Balearic Islands, Palma de Mallorca, Spain; ^2^Department of Infectious Diseases, Institute of Biomedicine, Sahlgrenska Academy, University of Gothenburg, Gothenburg, Sweden; ^3^Culture Collection University of Gothenburg (CCUG), Institute of Biomedicine, Sahlgrenska Academy, University of Gothenburg, Gothenburg, Sweden; ^4^Programa Institucional de Fomento a la Investigación, Desarrollo e Innovación, Universidad Tecnológica Metropolitana, Santiago, Chile; ^5^Center of Applied Ecology and Sustainability (CAPES), Santiago, Chile; ^6^Marine Biological Section, Department of Biology, University of Copenhagen, Helsingør, Denmark

**Keywords:** *Stutzerimonas balearica*, *Stutzerimonas*, *Pseudomonas stutzeri* group, comparative genomics, signature nucleotide positions, aromatic compounds biodegradation

## Abstract

*Stutzerimonas balearica* (*Pseudomonas balearica*) has been found principally in oil-polluted environments. The capability of *S. balearica* to thrive from the degradation of pollutant compounds makes it a species of interest for potential bioremediation applications. However, little has been reported about the diversity of *S. balearica*. In this study, genome sequences of *S. balearica* strains from different origins were analyzed, revealing that it is a diverse species with an open pan-genome that will continue revealing new genes and functionalities as the genomes of more strains are sequenced. The nucleotide signatures and intra- and inter-species variation of the 16S rRNA genes of *S. balearica* were reevaluated. A strategy of screening 16S rRNA gene sequences in public databases enabled the detection of 158 additional strains, of which only 23% were described as *S. balearica*. The species was detected from a wide range of environments, although mostly from aquatic and polluted environments, predominantly related to petroleum oil. Genomic and phenotypic analyses confirmed that *S. balearica* possesses varied inherent capabilities for aromatic compounds degradation. This study increases the knowledge of the biology and diversity of *S. balearica* and will serve as a basis for future work with the species.

## 1. Introduction

The species *Stutzerimonas balearica* (Gomila et al., 2022; Lalucat et al., 2022) (formerly, *Pseudomonas balearica* Bennasar et al., 1996) was proposed after the analysis and characterization of two denitrifying and salt-tolerant (i.e., growth in 8.5% NaCl, w/v) strains of the genomovar 6 of *Pseudomonas stutzeri* (currently, *Stutzerimonas stutzeri*) (Rossello et al., 1991; Rosselló-Mora et al., 1994; Bennasar et al., 1996; Lalucat et al., 2022), which had been isolated as 2-methylnaphthalene degrading strains from a wastewater treatment plant and from polluted marine sediment (Rossello et al., 1991). Since then, several strains of *Stutzerimonas balearica* have been reported through the years, such as *S. balearica* strain st101, isolated as a phenanthrene degrader from the rhizosphere of *Spartina patens* at an oil refinery site and reported also as a naphthalene degrader (Dutta, 2001; Mulet et al., 2008), *S. balearica* strain Z8, isolated from petrochemical waste water (Ahmadi et al., 2017), *S. balearica* strain EC28, isolated from corroded steel of a floating production storage and off-loading vessel, Salgar-Chaparro et al. (2020), and three strains which were isolated from a submarine volcano (Bravakos et al., 2021).

Previous reports indicate that *S. balearica* is a species with diverse properties and biodegradation capabilities, which is found principally in marine and polluted environments (Rossello et al., 1991; Bennasar et al., 1996; Dutta, 2001; Mulet et al., 2008; Ahmadi et al., 2017; Salgar-Chaparro et al., 2020; Bravakos et al., 2021). These observations make *S. balearica* a species of interest for bioremediation of areas polluted with recalcitrant compounds, such as aromatic hydrocarbons, some of which are among the most prevalent and persistent pollutants in the environment and are listed in the top 10 of the Substance Priority List of the Agency for Toxic Substances and Disease Registry [Agency for Toxic Substances and Disease Registry (ATSDR), Division of Toxicology and Human Health Sciences, 2019]. However, the heterogeneity of the former *P. stutzeri* group (also known as *P. stutzeri* complex) (Mulet et al., 2010), and the limited discriminatory power of the 16S rRNA gene, have frequently resulted in misclassifications and in strains not being identified at the species level (Lalucat et al., 2006; Gomila et al., 2022; Li et al., 2022; Uddin et al., 2022). For instance, routine protocols for identification, such as commercial API strips (BioMérieux, France), identify *S. balearica* strains as *S. stutzeri* (Uddin et al., 2022). This may result in *S. balearica* strains not being detected or being misidentified. An example is *S. balearica* YC-YH1, which was erroneously reported as *S. stutzeri* (Shi et al., 2015). Precise and accurate species identification is important because different taxonomic groups may have different capabilities and ecological adaptations (García-Valdés et al., 2010). For instance, *S. stutzeri* has been commonly reported in clinical samples; indeed a previous study (Scotta et al., 2012) reported that almost 80% of 138 analyzed clinical isolates of *Stutzerimonas* belonged to *S. stutzeri* (formerly, *P. stutzeri* genomovar 1), while no isolates were assigned to 11 of the 19 genomovars that had been described at that time, which suggests that different genomovars (i.e., phylogenomic species) of the genus *Stutzerimonas* may have different pathogenic potential. In the original description of *P. balearica* (currently, *S. balearica*), the authors defined 37 signature nucleotide positions of the 16S rRNA gene that differentiated *P. balearica* from the other six genomovars of *P. stutzeri* that had been described at that time (Bennasar et al., 1996). However, 15 additional genomovars and multiple closely-related species have been described since then. Therefore, it is unclear how many of these signature nucleotide positions are still relevant for identifying *S. balearica*. At this point, it is important to clarify that *S. balearica* is a member of the recently proposed genus *Stutzerimonas*, family *Pseudomonadaceae*, which includes species of the former *Pseudomonas stutzeri* phylogenetic group and currently comprises 51 phylogenomic species (Gomila et al., 2022; Lalucat et al., 2022).

The limitations in the detection and identification of *S. balearica* have hindered our understanding of the diversity, the biology and of the environmental distribution of the species. However, the development and the substantial reduction in the costs of whole-genome sequencing (Loman and Pallen, 2015) have resulted in a vast increase in the number of publicly available bacterial genome sequences (Sayers et al., 2022), which facilitates accurate species identification (Goris et al., 2007) and the exploration of the genomic diversity and physiological potential of species (Loman and Pallen, 2015).

The aim of this study was to analyze the genome sequences of strains of *S. balearica* from diverse sources and origins, together with the genome sequences of different phylogenomic species of *Stutzerimonas* and type strains of other species of the former *P. stutzeri* group to: firstly, explore the genomic diversity of *S. balearica* in its phylogenetic context; secondly, determine the current conservation and specificity of the 16S rRNA gene sequence signature nucleotide positions presented previously (Bennasar et al., 1996), thirdly, screen publicly available 16S rRNA gene sequences and determine the incidence and distribution of *S. balearica* at a larger scale; fourthly, determine the biodegradative capabilities of *S. balearica* toward aromatic compounds, based on genomic analyses and phenotypic testing.

## 2. Materials and methods

### 2.1. Strains and whole-genome sequences

A total of 18 genome sequences of *S. balearica* were included in the study, 11 derived from isolated strains and seven derived from metagenomic datasets (i.e., metagenome-assembled genomes, MAGs) (Supplementary Table 1). Of the 11 isolate-derived genome sequences, one was determined in this study (*S. balearica* strain SAGV3-2SA2 = CCUG 72049) and 10 were obtained from GenBank (Sayers et al., 2022), where nine were listed as *S. balearica* and one (that of strain YC-YH1) as *S. stutzeri*, although classified as *S. balearica* in the Genome Taxonomy Database (GTDB) (Parks et al., 2018). A recent study also confirmed this misclassification (Li et al., 2022).

MAGs of *S. balearica* were searched for in NCBI GenBank (Sayers et al., 2022) and GTDB (Parks et al., 2018). Additionally, a set of 52,515 published MAGs (Nayfach et al., 2021) was screened, using BLAST v2.9.0+ (Altschul et al., 1990) and the *rpoD* gene sequence of *S. balearica* DSM 6083^T^ as query sequence. The completeness and contamination levels of the MAGs were estimated, using CheckM v1.2.2 (Parks et al., 2015), implemented in DFAST_QC v.0.2.6 (Tanizawa et al., 2016). The searches resulted in seven MAGs of *S. balearica*, of which six were obtained from GenBank and one (*S. balearica* 3300027365_7) from the set of 52,515 MAGs (Nayfach et al., 2021). Of the six obtained from GenBank, two were classified as *S. balearica*, two as *S. stutzeri* and two as *Pseudomonas* sp., although all six were listed as *S. balearica* in GTDB.

Additionally, 76 genome sequences of strains listed as *S. stutzeri* in GenBank (Sayers et al., 2022) and the genome sequences of 19 additional type strains of species reported as members of the genus *Stutzerimonas* or the former *Pseudomonas stutzeri* group were also included in the study (Supplementary Table 1). Of these 19 additional type strains, five were listed originally as *S. stutzeri* in GenBank: the type strain of *S. stutzeri* and the type strains of the recently proposed species *Stutzerimonas perfectomarina*, *Stutzerimonas frequens*, *Stutzerimonas degradans*, and *“Stutzerimonas decontaminans*,” i.e., the former genomovars 2, 5, 7, and 4, respectively (Gomila et al., 2022; Mulet et al., 2023). Of the 113 genome sequences, 22 are complete genome sequences (including three *S. balearica*) and 91 are of draft status, with a number of contigs/scaffolds ranging from 1 to 298, among isolate-derived genome sequences, and ranging from 41 to 469 among MAGs.

### 2.2. DNA extraction and whole-genome sequencing

*S. balearica* strain SAGV3-2SA2 was cultivated on Columbia Blood Agar Base plus 5% defibrinated horse blood (Substrate Unit, Department of Clinical Microbiology, Sahlgrenska University Hospital, Gothenburg, Sweden), at 30°C, for 24 h. Genomic DNA was extracted, using a previously described protocol (Salvà-Serra et al., 2018). Subsequently, isolated DNA was used to prepare a standard Illumina library at Eurofins Genomics (Konstanz, Germany), following an optimized protocol and using standard Illumina adapter sequences. The library was sequenced, using an Illumina NovaSeq 6,000 system (Illumina, Inc., San Diego, CA, United States), to generate paired-end reads of 151 bp, distributed in 1.4 Gb.

### 2.3. Assembly and annotation of genome sequences

The Illumina sequence reads of *S. balearica* strain SAGV3-2SA2 were quality-evaluated, using FastQC 0.10.1 (Andrews, 2010), trimmed, using Sickle v1.33 (Joshi and Fass, 2011), and assembled *de novo*, using SPAdes v3.11.1 (Bankevich et al., 2012). The quality of the assembly was assessed, using QUAST v4.5 (Gurevich et al., 2013). The genome sequence was subsequently annotated, using the NCBI Prokaryotic Genome Annotation Pipeline (PGAP) v5.2 (Tatusova et al., 2016). All 106 genome sequences derived from isolated strains were downloaded from the NCBI Reference Sequence Database (RefSeq) (O'Leary et al., 2016), where they were annotated with PGAP. Of the seven MAGs, six were obtained from GenBank, annotated with PGAP. Of these, two were re-annotated on-line, using the DDBJ Fast Annotation and Submission Tool (DFAST) v1.2.6 (Tanizawa et al., 2017), since not all PGAP output files were available in GenBank. The other MAG (*S. balearica* 3300027365_7) was not available in GenBank (it was obtained from a previously published dataset of MAGs) (Nayfach et al., 2021) and, therefore, was also annotated, using DFAST. The DFAST annotations of these three MAGs were used for downstream functional annotations. The DFAST-generated GBFF files are available as Supplementary Files (Supplementary Files 1–3). Additionally, to normalize the annotations for the pan-genomic analyses, all 113 genome sequences were annotated, using Prokka v1.14.6 (Seemann, 2014).

### 2.4. Average nucleotide identity determinations

The average nucleotide identities based on BLAST (ANIb) (Goris et al., 2007) between all 113 genome sequences (all vs. all), were calculated, using JSpeciesWS (Richter et al., 2016). Calculations were done bidirectionally, and average values were determined. The ANIb values were used to construct a dendrogram and a heatmap, using Heatmapper, with an average linkage method for clustering and Pearson’s distance correlation (Babicki et al., 2016).

### 2.5. Phylogenomic species assignment

Two approaches were used to determine or confirm the species or phylogenomic species affiliations of the genome sequences included in this study. On the one hand, ANIb values were calculated between all the 113 genome sequences, which included multiple type strains and non-type reference strains of phylogenomic species of *Stutzerimonas*. Genome sequences sharing an ANIb value of greater than or equal to 95% were considered to belong to the same phylogenomic species. The phylogenomic species assignments were confirmed by calculating the digital DNA–DNA hybridization (dDDH) values between the assigned genome sequences and the genome sequences of the respective type or reference strains. The dDDH values were determined, using Genome-to-Genome Distance Calculator (GGDC) v3.0 (Meier-Kolthoff et al., 2013, 2022). On the other hand, a comparative analysis of partial *rpoD* gene sequences was performed, as previously described (Scotta et al., 2012). Briefly, the complete *rpoD* gene sequences were extracted from the genome sequences. The partial *rpoD* gene sequences of the reference strains of 21 phylogenomic species of *Stutzerimonas* (previously, the 21 genomovars of *S. stutzeri*), were downloaded from the PseudoMLSA database (Bennasar et al., 2010) and also included. *Pseudomonas aeruginosa* CCM 1960^T^ was used as outgroup. The *rpoD* sequences were aligned, using MUSCLE v3.8.425 (Edgar, 2004), implemented in AliView v1.25 (Larsson, 2014), and the distance matrix was built in MEGA7 (Kumar et al., 2016), using the Jukes-Cantor model (Jukes and Cantor, 1969). The phylogenetic tree was generated by neighbor-joining, bootstrap analysis (1,000 replications) and displayed, using iTOL (Letunic and Bork, 2021).

### 2.6. Core and pan-genome determinations

The protein FASTA files generated by Prokka v1.14.6 were used for proteome comparisons. All-versus-all proteome searches were performed, using BLASTP v2.9.0+. Protein clustering was performed, using the software GET_HOMOLOGUES v17112020 (Contreras-Moreira and Vinuesa, 2013), with three different algorithms: bidirectional best-hit (BDBH), COGtriangles (Kristensen et al., 2010) and Ortho Markov Cluster (OMCL) (Li et al., 2003). Two proteins were considered to belong to the same family if they showed at least 70% sequence identity over at least 70% of the length of the longest sequence in comparison. Based on the BDBH clustering, the core and pan-genome curves were represented and adjusted to the exponential models of Tettelin et al. (2005) and Willenbrock et al. (2007).

“Strict” consensus core genomes of single-copy orthologous sequences were determined, using the intersections of the three clustering algorithms, and used to construct phylogenomic trees. Briefly, the amino acid sequences of single-copy orthologous clusters were, first, independently aligned, using Clustal Omega v1.2.3 (Sievers et al., 2011). The resulting alignments were concatenated and processed, using Gblocks v0.91b to remove poorly aligned positions (Castresana, 2000; Talavera and Castresana, 2007). The resulting trimmed alignments were used to construct phylogenetic trees, using the software PhyML v3.1 (Guindon et al., 2010) and a maximum-likelihood estimation. A Shimodaira-Hasegawa-like approximate likelihood-ratio test (SH-aLRT) was used for branching statistical support (Anisimova and Gascuel, 2006). The phylogenomic trees were displayed, using iTOL (Letunic and Bork, 2021).

The pan-genomes were determined, using the intersections of clusters determined with COGtriangles and OMCL. The pan-genome clusters were classified according to their presence in all genomes (core), in 95% or more of the genomes (soft-core), in a large number of genomes but in less than 95% of them (shell), and present only in one or two genomes (cloud), as described previously (Koonin and Wolf, 2008; Contreras-Moreira and Vinuesa, 2013). The pan-genome matrix was used to determine the pan-genes of *S. balearica* (i.e., genes that are present in the 11 isolate-derived genome sequences of *S. balearica* and absent in all the 95 non-*S. balearica* genome sequences) (Contreras-Moreira and Vinuesa, 2013).

### 2.7. Evaluation of the 16S rRNA gene signature nucleotide positions of *Stutzerimonas balearica*

The 16S rRNA gene sequences were extracted from the isolate-derived genome sequences included in this study, using RNAmmer v1.2 (Lagesen et al., 2007), and aligned with the partial sequences of the reference strains of the former 21 genomovars of *P. stutzeri* (currently, phylogenomic species of *Stutzerimonas*), using MUSCLE v3.8.425 (Edgar, 2004), implemented in AliView v1.25 (Larsson, 2014). The seven MAGs analyzed did not contain any 16S rRNA gene sequence. The genome sequence of *S. balearica* YC-YH1 also did not contain any 16S rRNA gene sequence. Alternatively, a publicly-available and nearly-complete sequence was included (GenBank accession number: KJ786450.1). The genome sequences of some type strain genome sequences also lacked an assembled 16S rRNA gene sequence. In those cases, a 16S rRNA gene sequence was obtained from the List of Prokaryotic Names with Standing in Nomenclature (LPSN) (Parte, 2014). According to EzBiocloud, *Pseudomonas furukawaii* is the species presenting the highest 16S rRNA gene sequence similarity (97.8%) with that of *S. balearica*. Therefore, *P. furukawaii* (GenBank accession number AP014862.1), despite being a member of the *Pseudomonas resinovorans* group (Lalucat et al., 2020, 2022), was also included in the analysis.

Subsequently, the alignment was inspected manually to determine: first, if the 37 signature nucleotide positions are conserved among *S. balearica* strains; second, if the signature nucleotide positions are exclusive of *S. balearica*. Additionally, a distance matrix was constructed in MEGA7 (Kumar et al., 2016), using the Jukes-Cantor model (Jukes and Cantor, 1969), to determine the intra- and inter-species sequence variability of the 16S rRNA gene.

### 2.8. Search of 16S rRNA gene sequences of *Stutzerimonas balearica*

The partial 16S rRNA gene sequence of *S. balearica* DSM 6083^T^ (positions 50–650 bp, which encompass all 37 signature nucleotide positions; GenBank accession number: CP007511.1) was queried, using BLASTN, against the NCBI Nucleotide collection (nr/nt) database (Sayers et al., 2022), to find 16S rRNA gene sequences of additional strains of *S. balearica*. Sequences with similarities greater than or equal to 99.6% and greater than or equal to 90% of query coverage were downloaded and aligned, using MUSCLE v3.8.425 (Edgar, 2004), implemented in AliView v1.25 (Larsson, 2014). Sequences containing all 37 signature nucleotide positions of *S. balearica* were considered to be from members of the species.

### 2.9. Functional annotations of genome sequences

The protein sequences predicted by PGAP or DFAST (if PGAP was not available) were annotated, using Blast2GO (Conesa et al., 2005), InterProScan v5.54–87.0 (Jones et al., 2014) and eggNOG-Mapper v2.1.0 (Huerta-Cepas et al., 2017), with the eggNOG v5.0.2 database (Huerta-Cepas et al., 2019). Transfer of eggNOG annotations was limited to one-to-one orthology and to experimental evidence (i.e., prioritizing precision). The taxonomic scope was limited to *Gammaproteobacteria* (NCBI Taxonomy ID: 1236). The annotated Gene Onthology (GO) terms were validated to remove redundant terms based on the GO True Path Rule and filtered, using the class *Gammaproteobacteria* (Taxonomy ID: 1236). Subsequently, GOs were mapped to Enzyme Commission (EC) numbers, which were used together with EggNOG to annotate KEGG (Kyoto Encyclopedia of Genes and Genomes) pathways (Kanehisa et al., 2020). Blast2GO, EggNOG and InterProScan annotations were performed, using the platform OmicsBox v2.0.36 (BioBam Bioinformatics S.L., Valencia, Spain). The protein sequences were also classified into Clusters of Orthologous Groups (COG) categories (Galperin et al., 2015), using eggNOG-Mapper v2.1.6 (Huerta-Cepas et al., 2017) with the eggNOG v5.0 orthology resource (Huerta-Cepas et al., 2019), using the conditions listed above. Protein sequences of certain genes of interest were also analyzed, using InterPro 91.0 (Blum et al., 2020). Antibiotic resistance genes were searched, using the tool Resistance Gene Identifier (RGI) v5.2.1 of the Comprehensive Antibiotic Resistance Database (CARD) v3.1.4 (Alcock et al., 2020). Biocide and metal resistance genes were searched, using the BacMet v2.0 database (Pal et al., 2013) and the BacMet script *BacMet-Scan.pl*, against the database of “Experimentally confirmed resistance genes.” Virulence factors were searched, using VFanalyzer, of the Virulence Factors Database (VFDB) (Liu et al., 2022). Clustered regularly interspaced short palindromic repeats (CRISPR)-Cas systems were predicted, using the on-line tool CRISPRone (Zhang and Ye, 2017). Prophages were predicted, using PHASTER (Arndt et al., 2016). Putative integrative and conjugative elements (ICEs) and integrative and mobilizable elements (IMEs) were searched, using ICEfinder v1.0 (Liu et al., 2018). As ICEfinder can only analyze one single sequence per submission, the contigs or scaffolds of draft genome sequences and MAGs were merged into a single sequence per genome, using UGENE v43.0 (Okonechnikov et al., 2012), and contig/scaffolds were separated from each other by ten N’s. Natural transformation genes were searched for, using VFanalyzer (Liu et al., 2022), BLAST v2.5.0+ (Altschul et al., 1990), and the genome sequence of *Stutzerimonas nitrititolerans* strain DSM 10701 (= JM300; the former reference strain of the genomovar 8 of *S. stutzeri*) as a reference (Busquets et al., 2012). Genes encoding regulatory proteins were predicted, using P2RP v2.7 (Barakat et al., 2013).

### 2.10. *In silico* identification of pathways for degradation of aromatic compounds

Well-characterized gene products of key central and peripheral aromatic pathways (Ferrero et al., 2002; Pérez-Pantoja et al., 2008, 2012) were used as initial seeds to search for orthologous sequences throughout the genomes and MAGs of *S. balearica* strains, using BLAST v2.7.1 (Altschul et al., 1990). Proteins displaying at least 30% amino acid identity with query sequences were selected for additional analysis, including sequence similarity search in the protein database of GenBank (Sayers et al., 2022), identification of conserved domains, using the Conserved Domain database v3.19 (Lu et al., 2019), and manual inspection of gene neighborhoods, using Artemis v17.0.1 (Carver et al., 2011). Once a key orthologous sequence of central and/or peripheral aromatic pathway was identified, a complementary gene search of the whole pathway was performed, using the same strategy as before. A schematic representation of presence/absence of genes was generated, using Heatmapper (Babicki et al., 2016).

### 2.11. Growth assays of aromatic compounds

The six strains of *S. balearica* available at the Culture Collection University of Gothenburg (CCUG) were grown in mineral salts medium (14 g/l Na_2_HPO_4_·12H_2_O, 2 g/l KH_2_PO_4_, 50 mg/l Ca(NO_3_)_2_·4H_2_O, 1 g/l (NH_4_)_2_SO_4_, 200 mg/l MgSO_4_·7H_2_O, 278 μg/L FeSO_4_·7H_2_O, 70 μg/L ZnCl_2_, 100 μg/L MnCl_2_·4H_2_O, 62 μg/L H_3_BO_3_, 190 μg/L CoCl_2_·6H_2_O, 17 μg/L CuCl_2_·2H_2_O, 24 μg/L NiCl_2_·6H_2_O, 36 μg/L Na_2_MoO_4_·2H_2_O), supplemented with 2.5 mM benzoate, phenol, salicylate, naphthalene, phenylalanine and tyrosine as sole carbon and energy source. Naphthalene was dissolved in acetone. As growth control, acetone was used without the addition of naphthalene. The cultures were incubated in borosilicate glass tubes at 30°C for 5 days and optical densities at 600 nm (OD_600_) were measured, using a Synergy HTX multimode plate reader (BioTek, Winooski, VT, United States). Cultures were inoculated with 100-fold dilutions from overnight cultures grown on R2A broth. At least three biological replicates were performed for each growth measurement.

## 3. Results and discussion

### 3.1. ANIb and phylogenomic species assignment of genome sequences

**Figure 1:**
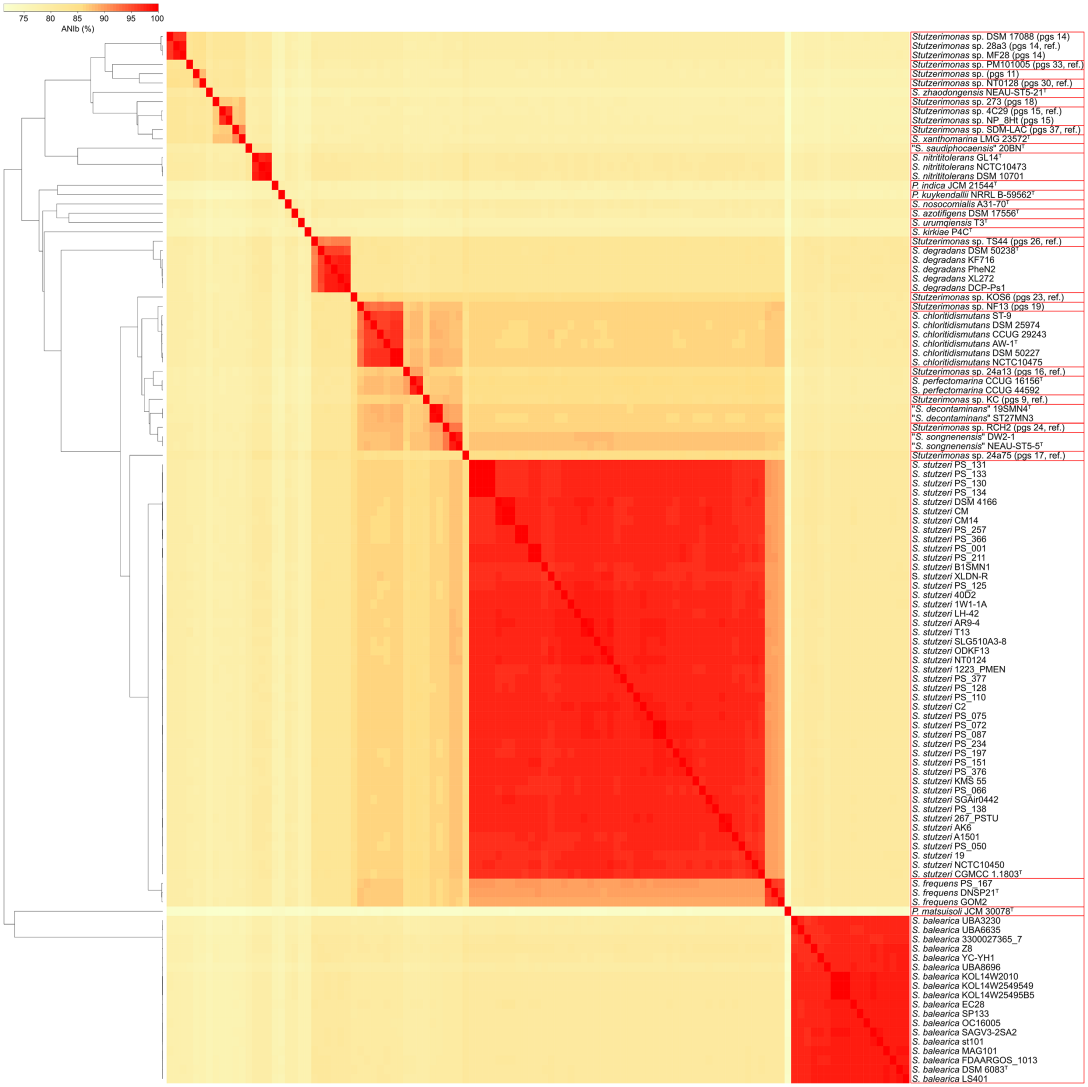
Dendrogram and heatmap, based on the ANIb values calculated between all 113 genome sequences included in this study. The phylogenomic species affiliations are indicated in parenthesis. Red squares indicate clusters of strains sharing greater than or equal to 95% of ANIb value (i.e., same genomic species). Pgs, phylogenomic species; ref., reference.

The ANIb values were calculated between all the 113 genome sequences included in this study (Supplementary Table 2), which revealed a total of 33 genomic species clusters and confirmed the species identities of the 18 genome sequences of *S. balearica* (Figure 1). It is notable that, of the 16 confirmed genome sequences of *S. balearica* obtained from GenBank, three were misclassified as *S. stutzeri* and two were listed as *Pseudomonas* sp., which exemplifies the problem of the poor classifications or misclassifications of strains of *S. balearica*. The lowest ANIb value between *S. balearica* strains was 96.54%, while the highest was 100%, i.e., between strains KOL14W2010, KOL14W2549549, and KOL14.W25.495.B5. These three strains were isolated from the same geographical area at Kolumbo submarine volcano (Greece), which probably explains the high level of genomic sequence similarity (Mandalakis et al., 2019). When excluding the values between these clonal strains, the highest ANIb value within *S. balearica* was 98.46%. Meanwhile, the highest ANIb between *S. balearica* DSM 6083^T^ and a non-*S. balearica* strain of the dataset of this study was observed to be 81.24% (*Stutzerimonas* sp. strain 24a75, pgs 17, ref.), and the highest ANIb value with a non-*S. balearica* type strain was with *S. degradans* DSM 50238^T^ (81.08%), followed by *S. stutzeri* CGMCC 11803^T^ (80.93%). Among other strains of *Stutzerimonas* species, the highest inter-pgs ANIb value was 92.51% (between *Stutzerimonas chloritidismutans* AW-1^T^, and *Stutzerimonas* sp. strain NF13, pgs 19), while the lowest was 74.85% (*Stutzerimonas urumqiensis* T3^T^, and *Stutzerimonas zhaodongensis* NEAU-ST5-21^T^).

The ANIb values between the 20 type strains included in this study ranged from 71.24% (*S. zhaodongensis* NEAU-ST5-21^T^ and *Pseudomonas matsuisoli* JCM 30078^T^) to 95.84% (*Stutzerimonas kunmingensis* DSM 25974^T^ and *S. chloritidismutans* AW-1^T^). The high ANIb value indicates that *S. kunmingensis* and *S. chloritidismutans* are, in fact, synonymous, which is in agreement with previous observations (Hesse et al., 2018; Lalucat et al., 2020). Indeed, Gomila et al. emended the description of *S. chloritidismutans,* which includes strains of former *P. stutzeri* genomovar 3, of which *S. kunmingensis* is a later heterotypic synonym (Cladera et al., 2006; Gomila et al., 2022). This is supported also in this study by the high ANIb values (>96%) between the type strains of *S. chloritidismutans*, *S. kunmingensis* and the reference strain of the former *P. stutzeri* genomovar 3. If the synonyms are excluded, the highest ANIb value between type strains of *Stutzerimonas* is 89.29% (between *S. frequens* DNSP21^T^ and *S. stutzeri* CGMCC 11803^T^). Additionally, when looking at the ANIb values between the 20 type strains, one notices that *P. matsuisoli* exhibits the lowest values. This species was considered to be a member of the *P. stutzeri* group by Lalucat et al. after performing a four-gene multilocus sequence analysis (MLSA) (Lalucat et al., 2020). However, *P. matsuisoli* was the most distantly-related species, together with *Pseudomonas indica* and *Pseudomonas kuykendallii*, which were previously also considered to be members of the former *P. stutzeri* group (Lalucat et al., 2020). Indeed, these three species have been recently shown to not be members of the former *P. stutzeri* group and to form ‘orphan’ groups of individual single species (Girard et al., 2021; Lalucat et al., 2022).

Worth noting is that *S. stutzeri* strain DSM 17088 was supposed to be the reference strain of pgs 12 (Sikorski et al., 2005; Gomila et al., 2022), but instead was affiliated with pgs 14. Partial *rpoD* sequencing of the CCUG deposit of this strain (i.e., CCUG 50543) revealed the same problem (GenBank accession number: OP081805.1). This suggests that a mix-up probably happened before the strain was deposited in these two culture collections. The ANIb analysis also confirmed that strain DW2-1 is a member of *“Stutzerimonas songnenensis*,” which is supported by the *rpoD* comparative analysis (Supplementary Figure 1) and in agreement with Li et al. (2022).

The inclusion of partial *rpoD* sequences of all the reference strains of the 21 former *P. stutzeri* genomovars allowed the assignment of two strains to phylogenomic species for which the genome sequences for the reference strains were not available for this study. Partial *rpoD* data also indicated that *S. zhaodongensis* is equivalent to the former genomovar 20, which supports the results from Gomila et al. (2022). However, in three cases, the *rpoD* analysis contradicted the ANIb results. One of these cases was the type strain of *S. kunmingensis* (DSM 25974^T^), which is synonymous with *S. chloritidismutans*, according to the ANIb analyses, but was assigned to *Stutzerimonas* pgs 19 by the *rpoD* analysis. Two other cases were the strains PS_066 and SGAir0442, which, according to ANIb analyses are members of *S. stutzeri*, but clustered with strain KOS6 (pgs 23, ref.) in the *rpoD* analysis. For the rest of strains, the *rpoD* analysis was in agreement with the ANIb results, although the observed discrepancies highlight the importance of relying, when possible, on whole-genome comparisons rather than single gene analyses.

In total, 75 of the 81 strains originally classified as *S. stutzeri* in GenBank were assigned to 15 of the 21 former *P. stutzeri* genomovars and one strain was assigned to *“S. songnenensis*.” These 75 strains included two *S. perfectomarina* strains (including the type strain), four *S. chloritidismutans* strains (including the type strain), three *S. frequens* strains (including the type strain), five *S. degradans* strains (including the type strain), two *S. nitrititolerans* strains, and two *“S. decontaminans*” strains (including the type strain), i.e., the former genomovars 2, 3, 5, 7, 8 and 4, respectively (Gomila et al., 2022; Mulet et al., 2023). The remaining six strains had been assigned previously to recently proposed phylogenomic species of *Stutzerimonas* (Gomila et al., 2022).

### 3.2. Core and pan-genome analyses of *Stutzerimonas balearica*

**Figure 2:**
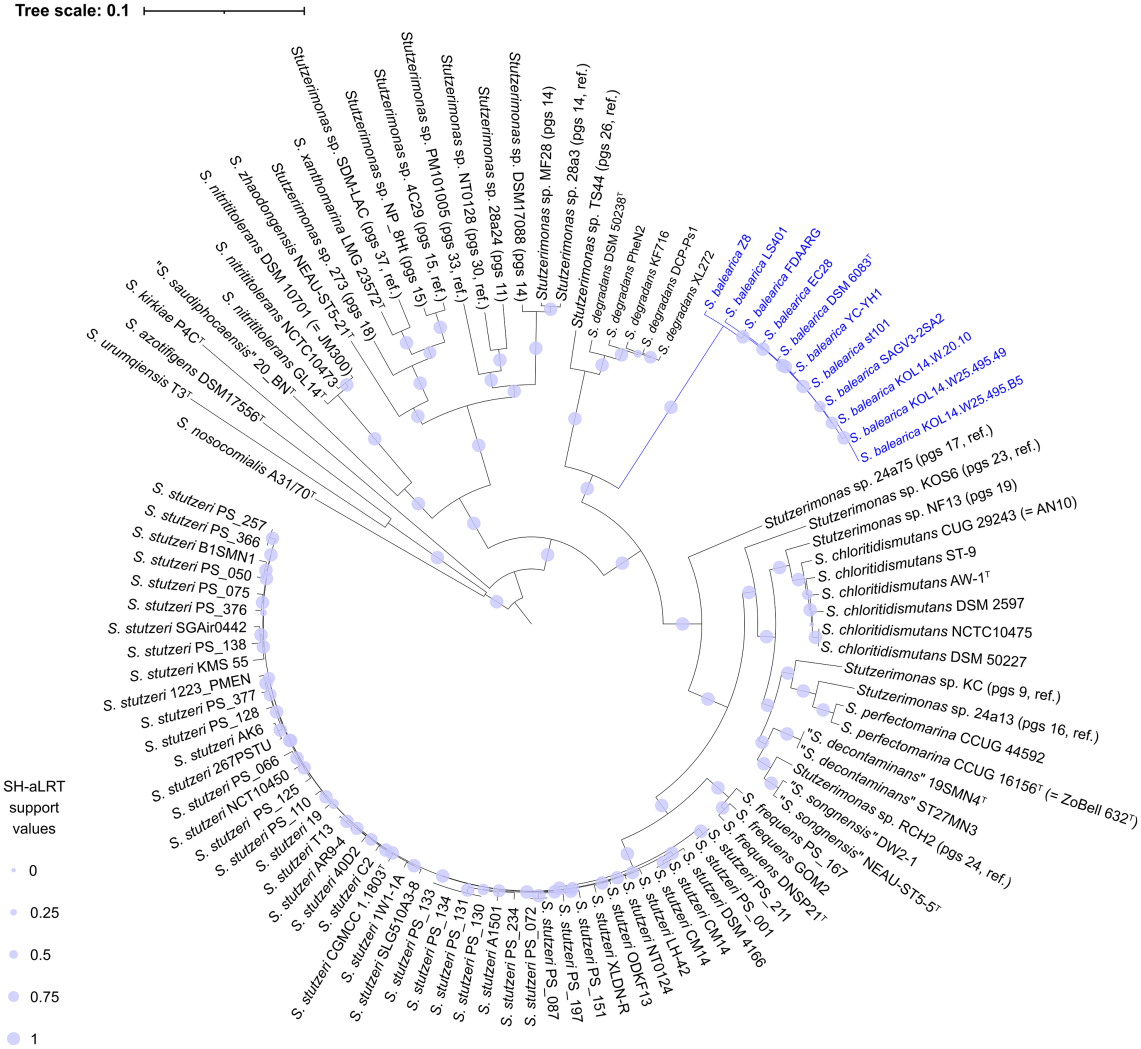
Core genome-based phylogenomic tree of the 11 isolate-derived genome sequences of *S. balearica* and 92 strains of other species and phylogenomic species of the genus *Stutzerimonas*. The tree was generated, based on 210,488 homologous amino acid positions, derived from 699 single copy core proteins. Pgs, phylogenomic species; ref., reference.

A pan-genome analysis was carried out, including the Prokka-annotated protein sequences of the 113 genome sequences used in the ANIb calculations. The analysis revealed a “strict” consensus core genome of single copy proteins, which was formed by 535 proteins when excluding the seven MAGs and 699 proteins when excluding the type strains of *P. indica*, *P. kuykendallii* and *P. matsuisoli*. These three type strains were reported previously to be members of the *P. stutzeri* group (i.e., *Stutzerimonas*) after a four-gene MLSA (Lalucat et al., 2020), but later shown to form orphan groups, by phylogenomic analyses (Girard et al., 2021; Lalucat et al., 2022). The observed numbers are in line with the 524 single-copy orthologous genes shared between the 123 strains of the *P. stutzeri* complex and the four outgroup strains analyzed by Li et al. (2022). These are also in accordance with the 666 core genes detected by Gomila et al. when outgroup strains of the family *Pseudomonadaceae* were included with the analysis of 200 strains (Gomila et al., 2022). The 699 proteins, totaling 210,488 amino acid positions, were used to construct a phylogenomic tree (Figure 2). In this 103-genomes phylogenomic tree, *S. balearica* strains cluster together with strains of *S. degradans* and *Stutzerimonas* sp. strain TS44 (pgs 26, ref.), but forming a distinct and separate branch. A second phylogenomic tree was also constructed, using a “strict” consensus single-copy core genome of 127 protein sequences, totaling 35,374 amino acid positions, including the MAGs and the type strains of *P. indica*, *P. kuykendallii* and *P. matsuisoli* (Supplementary Figure 2). The two trees showed highly similar structures and, in both cases, all strains of *S. balearica* clustered together in a single monophyletic branch, closely-related to *S. degradans* and *Stutzerimonas* sp. strain TS44. The 113-genomes tree also shows that the type strains of *P. indica*, *P. kuykendallii* and *P. matsuisoli* were the three strains most distantly related from the type strain of *S. stutzeri*, i.e., the type species of the genus *Stutzerimonas*, which confirms the results from Girard et al. (2021) and Lalucat et al. (2022).

**Figure 3:**
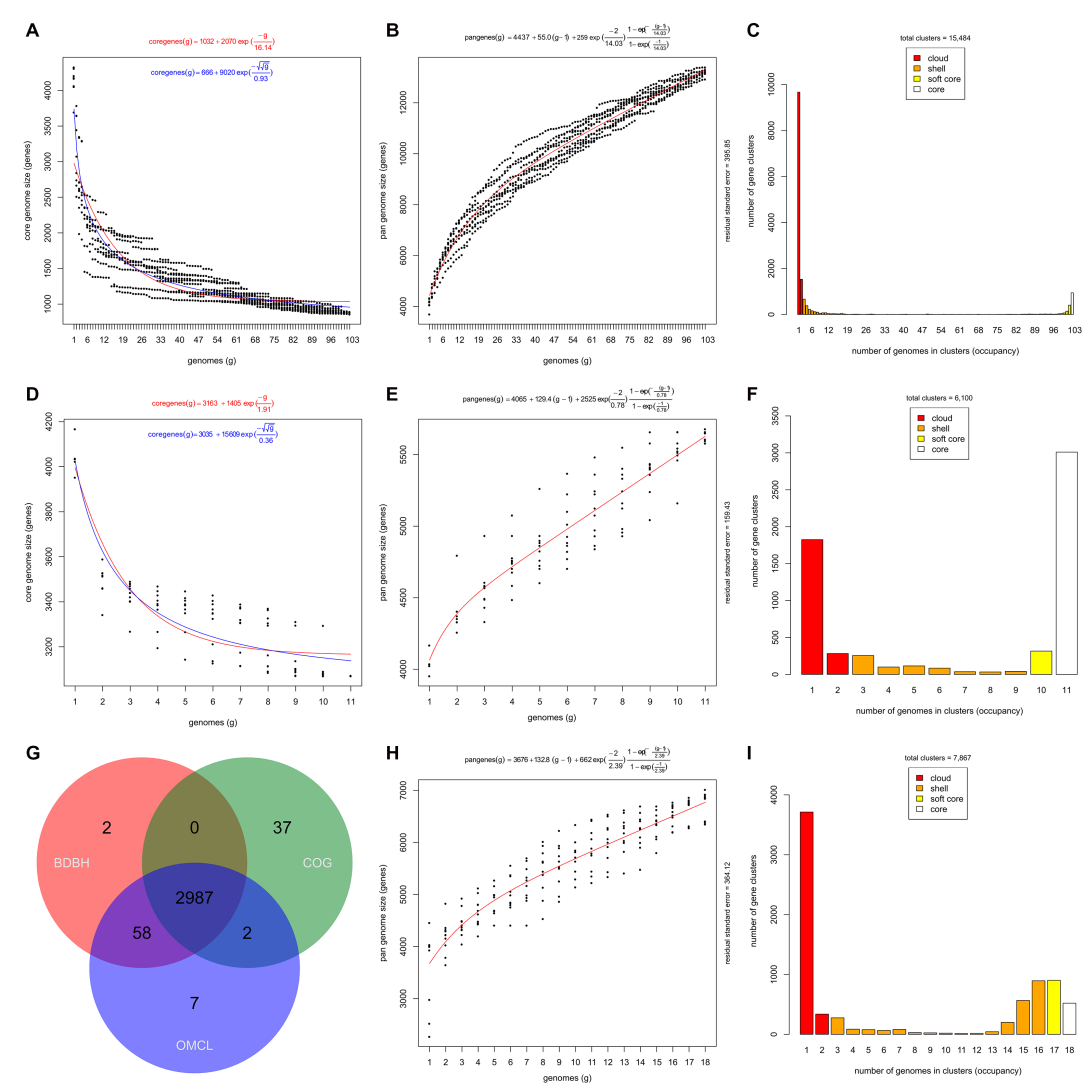
Core and pan-genome representations of *Stutzerimonas* and *S. balearica*. **(A)** Estimate of the core genome size of the 103 isolate-derived *Stutzerimonas* genome sequences fitted to the Tettelin (red) and Willenbrock (blue) exponential decay models. **(B)** Estimate of the pan-genome size of the 103 isolate-derived *Stutzerimonas* genome sequences fitted to the Tettelin exponential model. **(C)** Distribution of the OMCL-COGtriangles pan-genome matrix of the 103 isolate-derived *Stutzerimonas* genome sequences into cloud, shell, soft core, and core compartments. **(D)** Estimate of the core genome size of the 11 isolate-derived genome sequences of *S. balearica*. **(E)** Estimate of the pan-genome size of the 11 isolate-derived genome sequences of *S. balearica*. **(F)** Distribution of the OMCL-COG triangles pan-genome matrix of the 11 isolate-derived genome sequences of *S. balearica*. **(G)** Venn diagram of the core genomes generated by the three clustering algorithms: BDBH, COG triangles, and OMCL. **(H)** Estimate of the pan-genome size of the 11 isolate-derived genome sequences and the seven MAGs of *S. balearica*. **(I)** Distribution of the OMCL-COGtriangles pan-genome matrix of the 11 isolate-derived genome sequences and seven MAGs of *S. balearica*.

The BDBH clustering algorithm estimated a core genome size of nearly 900 gene clusters (estimations ranging from 858 to 893 gene clusters) for the 103 isolate-derived genome sequences of *Stutzerimonas* (i.e., the seven MAGs and the three non-*Stutzerimonas* genomes excluded) (Figure 3A). The Tettelin and the Willenbrock models estimated the size of the core genome of the 103 genome sequences in 1,035 and 957 gene clusters, respectively (Figure 3A). If 1,000 were added, according to these models, the sizes of the core genome would be 1,032 and 687 gene clusters, respectively. These estimations are in line with the 1,104 and 1,054 core genes predicted after including 123 (27 genomic species) (Li et al., 2022) and 200 genome sequences (45 genomic species) (Gomila et al., 2022), respectively. The core genome estimations may vary depending on the algorithms used, clustering parameters, the number of strains, their diversity and the quality of the genome sequences and annotation, but all in all, demonstrate that the members of the genus *Stutzerimonas* share a relatively small core genome, which represents approximately a 15–25% of the average number of coding sequences per genome annotated by PGAP in the *Stutzerimonas* genomes included in this study. This is in line with what has been found among other members of the family *Pseudomonadaceae* (Udaondo et al., 2016; Gomila et al., 2017; Freschi et al., 2019; Whelan et al., 2021). However, larger scale analyses including more genomes (and if possible complete) and more genomic species are necessary to get more exact estimations. For instance, after the inclusion of more than 1,300 genome sequences, Freschi et al. determined that the core genome of *P. aeruginosa* was formed by 665 genes, which represents approximately 10% of a typical *P. aeruginosa* genome (Freschi et al., 2019).

The analysis also revealed that the genome sequences derived from 103 isolated strains of *Stutzerimonas* species included in this study exhibit an open pan-genome (Figure 3B), formed by 15,484 gene clusters, according to the estimation of the OMCL-COGtriangles algorithms (Figures 3B,C). When the seven MAGs of *S. balearica* were included, the size increased to 16,628 gene clusters. These pan-genome sizes are in line with the studies published by Li et al. (2022) and Gomila et al. (2022), which reported 13,261 and 18,654 gene clusters, respectively. The Tettelin exponential model estimated that if 1,000 genomes were analyzed, the pan-genome would comprise 62,647 gene clusters, which indicates that the isolation and sequencing of additional strains of *Stutzerimonas* will reveal large amounts of novel genes. However, large-scale studies, such as that of Freschi et al. (2019) are needed to get more precise estimations. Moreover, studies aiming to sequence novel strains of *Stutzerimonas* would probably end up revealing novel genomic species and, thus, increase the knowledge of the diversity of *Stutzerimonas*. It is also notable that nearly 10,000 of the 15,484 gene clusters were found in only one strain (Figure 3C), which confirms the extremely high diversity of the genus *Stutzerimonas*.

The analysis of the 11 isolate-derived genome sequences of *S. balearica* revealed a core genome of 3,071 gene clusters (BDBH estimations) (Figure 3D). The estimated core genome sizes, according to the Tettelin and Willenbrock models, were 3,167 and 3,138 gene clusters, respectively. According to these models, the sizes of the core genome would decrease to 3,163 and 3,037, respectively, if 1,000 genomes were analyzed. In any case, this should be confirmed by including additional strains of *S. balearica* in the analyses. The analyses also revealed that *S. balearica* has an open pan-genome (Figure 3E), formed by 6,100 gene clusters according to the OMCL-COGtriangles algorithms (Figure 3F). The consensus “strict” single-copy core genome (i.e., the intersection of the BDBH, COG and OMCL algorithms) was formed by 2,987 gene clusters (Figure 3G). When the MAGs were included, the size of the pan-genome estimated by the OMCL-COGtriangles algorithms increased to 7,867 gene clusters (Figures 3H,I). In the analysis of the 11 isolate-derived genome sequences of *S. balearica*, the Willenbrock model estimated that, if 100 genomes were analyzed, the pan-genome would be formed by 17,144 gene clusters. If the seven MAGs were included in the analysis, the predicted pan-genome for 100 genome sequences increased slightly to 17,661 gene clusters. This small increase, which occurred despite the relatively low completeness of four of the MAGs, might be due to contamination during the binning process, even if CheckM indicated low levels of contamination (<3%) (Parks et al., 2015). More contiguous or even complete MAGs with less contamination could be obtained by using long-read sequencing in metagenomic studies (Chen et al., 2020; Sereika et al., 2022). The presence of the three clonal strains in the pan-genomic analysis may also have an effect on the exponential models and cause an underestimation of the slope of the pan-genome, but in any case, these results indicate that *S. balearica* has a clearly open pan-genome and that sequencing additional strains will continue revealing new genes.

The pan-genome analyses also revealed 24 genes which were present in all of the 11 isolate-derived genome sequences of *S. balearica* but absent in the 95 non-*S. balearica* genome sequences (i.e., pan-genes; Supplementary Table 4). Annotations revealed that 17 of the genes encode membrane-bound proteins. Four and seven of the genes were grouped in two respective gene clusters. In the first gene cluster, two genes encode hypothetical proteins that could not be assigned to any function, another one a transcription factor, and a fourth one an EAL domain-containing protein, which could potentially serve as a phosphodiesterase or regulate cell motility and biofilm formation (Youssef et al., 2017). The same gene cluster also contains a gene encoding a carbon storage regulator protein, which is not exclusive of *S. balearica*. The second gene cluster includes genes encoding subunits of a NADH-quinone oxidoreductase. In *P. aeruginosa*, the presence of three enzymes for catalyzing the NADH:quinone oxidoreductase step of the respiratory chain, which confer resilience on its energy production systems, was shown in a previous study (Hreha et al., 2021). Another pan-gene, which is not located in the NADH-quinone oxidoreductase gene cluster, also encodes a protein potentially involved in the electron transport chain, possibly a cytochrome C oxidase subunit. Other genes encode proteins with domains for various activities. These include a methyltransferase domain, a protein with an acetyltransferase domain, and possible S-methyl-5-thioribose-1-phosphate isomerase activity. Another gene encodes a dTDP-4-dehydrorhamnose reductase, potentially involved in dTDP-L-rhamnose biosynthesis and thus in the synthesis of lipopolysaccharide and rhamnolipids (i.e., surfactants) (Olvera et al., 1999). Finally, a gene codifies for a membrane lipoprotein and multiple genes encode hypothetical proteins.

### 3.3. Clusters of orthologous groups functional annotation of *Stutzerimonas balearica*

**Figure 4:**
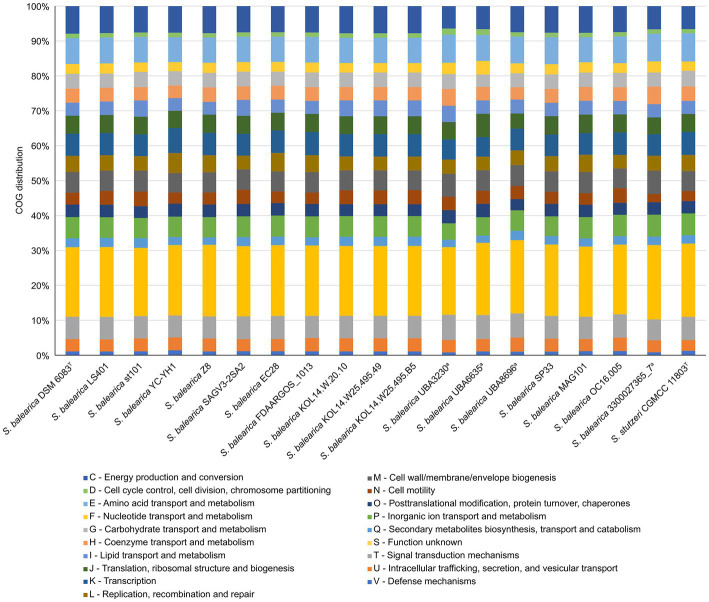
COG functional categories distribution of the accessioned proteins of the isolate-derived genome sequences and MAGs of *S. balearica* and the type strain of *S. stutzeri*. ^a^MAGs with a CheckM completeness estimate lower than 95%.

The accessioned protein sequences annotated by PGAP or DFAST were assigned to COG functional categories. The distribution of the COG categories was similar among all the *S. balearica* strains and, in all of them, about a 20% of the proteins were assigned to the category “function unknown” (Figure 4), highlighting once again the need for studies to characterize and unveil the function of yet undescribed proteins. Other categories with large numbers of assigned proteins were “energy production and conversion,” “amino acid transport and metabolism,” and “inorganic ion transport and metabolism,” which might be due to the wide metabolic versatility of *Stutzerimonas* (Lalucat et al., 2006). The categories “signal transduction mechanisms” and “transcription” were also among those with the highest numbers of assigned proteins, which is in line with the large regulatory network previously reported in *Pseudomonas* (Stover et al., 2000). The category “cell wall/membrane/envelope biogenesis” also encompassed numerous proteins (>200 per strain), which is reasonable, considering that *S. balearica* is a widely distributed and versatile species that deals with numerous environmental conditions and is exposed to a wide range of stressing conditions.

### 3.4. Habitats and environmental distribution of *Stutzerimonas balearica*

The description of *S. balearica*, presented 37 species-specific 16S rRNA gene signature nucleotide positions distributed in 18 regions of the 16S rRNA gene (Bennasar et al., 1996). Those signatures were determined by comparing the 16S rRNA gene sequences of 2 *S. balearica* strains with the consensus 16S rRNA gene sequences of the former *P. stutzeri* strains, which, at that time, were classified in six genomovars. However, since then, many species and phylogenomic species of *Stutzerimonas* and species of *Pseudomonas* have been described and more strains of *S. balearica* obtained. For these reasons, the current relevance of the described signature nucleotide positions for detecting and differentiating strains of *S. balearica* is unclear.

**Figure 5:**
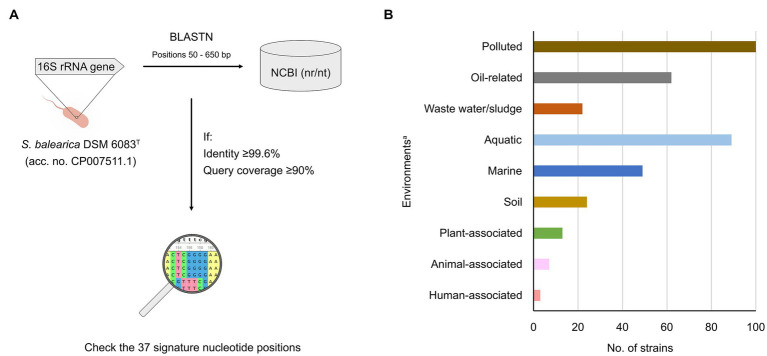
Search strategy and origin of strains of *S. balearica*. **(A)** Two-step strategy to detect and identify public 16S rRNA gene sequences of *S. balearica* strains. The 16S rRNA gene sequence of *S. balearica* DSM 6083^T^ (positions 50–650 bp) is analyzed, using BLASTN. The signature nucleotide positions are checked in sequences showing ≥99.6% of sequence identity and ≥ 90% of query coverage. **(B)** Sources of the 164 *S. balearica* strains for which source information was available. ^a^The strains were assigned to one or more environments, based on the source information.

The comparison of the sequences included in this study revealed that the highest 16S rRNA gene sequence identity between *S. balearica* DSM 6083^T^ and the type strain of another species was 97.7% (*P. furukawaii* KF707^T^, in agreement with EzBiocloud), while the lowest sequence identity among strains of *S. balearica* was 99.6%. The analysis also revealed that only one signature position remains exclusive to *S. balearica* (*E. coli* position 224). This was partly due to the relatively high similarities of the 16S rRNA gene sequences of the type strain of *S. balearica* and the type strains of *S. azotifigens*, *P. furukawaii*, *P. indica*, *P. matsuisoli*, *S. urumqiensis*, particularly in the hypervariable region V1, which harbors 18 of the 37 signature nucleotide positions. However, none of the non-*S. balearica* strains contained the combination of all 37 signature nucleotide positions, while the 16S rRNA gene sequences derived from all 10 *S. balearica* genomes and the nearly-complete sequence of strain YC-YH1 maintained all 37 signature nucleotide positions. These results indicate that the 16S rRNA gene sequences can be used effectively to differentiate *S. balearica* from other closely-related species. This allowed us to establish a two-step strategy to screen for and detect strains of *S. balearica* in public sequence databases, based on sequence identity and identification of the 37 signature nucleotide positions (Figure 5A).

The BLASTN of the 16S rRNA gene sequence of *S. balearica* DSM 6083^T^ against NCBI Nucleotide collection (nr/nt) database yielded 173 sequences with 99.6% or greater sequence identity and with 90% or greater query coverage. Of these, 158 were strains without publicly available genome sequence that contained all 37 signature nucleotides. All of these sequences were considered to be derived from authentic *S. balearica* strains that, together with the 18 that were already included in the study, add up to as many as 176 *S. balearica* strains (Supplementary Table 3). This demonstrates that the proposed strategy is a useful method for identifying strains of *S. balearica* by analyzing 16S rRNA gene sequence in cases where genomic data is not available. To increase further the reliability of the identification, the strategy could be complemented with *rpoD* gene sequencing, which despite the discrepancies that have been observed for a few strains of some species of *Stutzerimonas*, has shown full agreement with the genomic-based taxonomic assignments of *S. balearica*.

The strategy is highly restrictive, as it allows only for strains exhibiting sequences with 99.6% or greater identity and all 37 signature nucleotide positions, to be considered as authentic *S. balearica*. A consequence of this is that authentic *S. balearica* strains probably have been missed, because their partial 16S rRNA gene sequences did not cover one or more signature nucleotide positions. However, we consider that being strict and missing strains is more reliable than setting relaxed thresholds and having a higher risk of obtaining false positives.

It is notable that only 23% (37/158) of the sequences were originally labeled as *S. balearica*, while 68% (107/158) were assigned to higher rank taxa and 9% (14/158) were misclassified, 13 as *S. stutzeri* and one as *Mesobacillus subterraneus*. This shows, once again, the problems with lack of accuracy and misclassified sequences in public databases (Ashelford et al., 2005; Yarza et al., 2008), which is widely extended and was even shown to affect specialized 16S rRNA gene sequence databases, such as SILVA and RDP (Ribosomal Database Project) (Edgar, 2018), despite the availability of tools and curated databases, such as EzBiocloud, that can help to alleviate the problems of inaccurate and wrongly classified of 16S rRNA gene sequences (Yoon et al., 2017).

Information about the geographical origins was available for 116 of the 176 *S. balearica* strains (Supplementary Table 3). These strains, alone, cover more than 30 countries from around the world and illustrate the geographically ubiquitous distribution of *S. balearica*. Additionally, information about the sources was available for 164 of the strains and included a wide range of environments (Figure 5B), including diverse aquatic and terrestrial ecosystems and several plant, animal, and fungal hosts. Interestingly, 61% (100 of 164) originated from polluted or anthropogenic environments, which is in agreement with what previous reports have suggested (Rossello et al., 1991; Bennasar et al., 1996; Dutta, 2001; Mulet et al., 2008; Ahmadi et al., 2017; Salgar-Chaparro et al., 2020). Moreover, 13% (22 of 164) originated from waste waters or sludge and 38% (62 of 164) originated from petroleum oil-related sources. This could be due to sampling bias toward these kinds of environments or a selection for *S. balearica*, but, in any case, such a predominance of suchlike environments highlights the relevance and the potential of *S. balearica* strains for bioremediation applications.

Additionally, 54% of these strains (89 of 164) were obtained from aquatic or water-containing sources and, 55% of these (49 of 89), originated from marine environments, which is in accordance with the described capacity of *S. balearica* to grow in the presence of 8.5% NaCl (Bennasar et al., 1996). Moreover, Li et al. recently suggested that the ancestors of the *P. stutzeri* complex may have originated from high-osmolarity environments, due to the presence of an ectoine biosynthesis gene cluster present in all genomes of the *P. stutzeri* complex (currently *Stutzerimonas*) and absent in most of the other *Pseudomonas* genomes (Li et al., 2022). Meanwhile, 13 strains were associated with plants, one with fungal spores, seven with animals and, interestingly, three were derived from human samples, one from the skin of a healthy individual and two from clinical samples. Indeed, a recent study reported a clinical carbapenem-resistant *S. balearica* strain isolated from a tracheal aspirate and carrying a VIM metallo-β-lactamase (Uddin et al., 2022). This suggests that potential of *S. balearica* to cause opportunistic infections and to carry relevant antimicrobial-resistance factors should not be disregarded, especially considering that rapid phenotypic identification methods often misidentify *S. balearica* strains as *S. stutzeri* (Uddin et al., 2022). Indeed, *S. stutzeri* is often isolated from clinical samples (Köse et al., 2004; Sader and Jones, 2005; Scotta et al., 2012; Mulet et al., 2017), which highlights the importance of accurate species and phylogenomic species identification in the clinics. Altogether, these data demonstrate that *S. balearica* is a versatile bacterium that can be found in a wide range of environments, similar to that of the nearly universal presence of *S. stutzeri* and *P. aeruginosa* (Lalucat et al., 2006; Crone et al., 2019). In many cases, *S. balearica* strains have been isolated because of certain metabolic and physiological properties of interest, such as denitrification, oxidation of thiosulfate, oxidation of 2-chloroethanol and iron, halotolerance, biofilm formation, antimicrobial properties, degradation of pesticides and polyethylene, biosurfactant production, but especially for capacities for biodegradation of aromatic hydrocarbons (Supplementary Table 3). Additionally, several studies have reported on strains of *S. balearica* with other properties of interest, such as amylase production (Kizhakedathil and Subathra, 2021), nitrogen fixation (Beauchamp et al., 2006), degradation of tributyltin (used as a biocide in antifouling paints) (Sampath et al., 2012), methylmercury decomposition (Lee et al., 2012) or bioleaching of gold and silver (Kumar et al., 2018). However, the identifications of such strains could not be verified or did not fulfill the identification thresholds used in this study. Altogether, these observations reinforce the idea that *S. balearica* is a species with large potential, not limited to bioremediation applications, particularly in highly-polluted marine and salty environments.

### 3.5. Degradation of aromatic compounds by *Stutzerimonas balearica*

Strains of *Stutzerimonas* are well known for their potential in aerobic bioremediation of aromatic compounds (Lalucat et al., 2006) and, indeed, several strains of *S. balearica* have been reported to be degraders of aromatic compounds (Rossello et al., 1991; Dutta, 2001; Parreira et al., 2011). To draw a wider picture of the potential of *S. balearica* for bioremediation, gene products of central and peripheral pathways of catabolism of aromatic compounds were searched for in the 11 isolate-derived genome sequences and seven MAGs. Additionally, growth assays to confirm their degradative capabilities were performed on six of these 11 strains (i.e., the six that were available at the CCUG).

**Figure 6:**
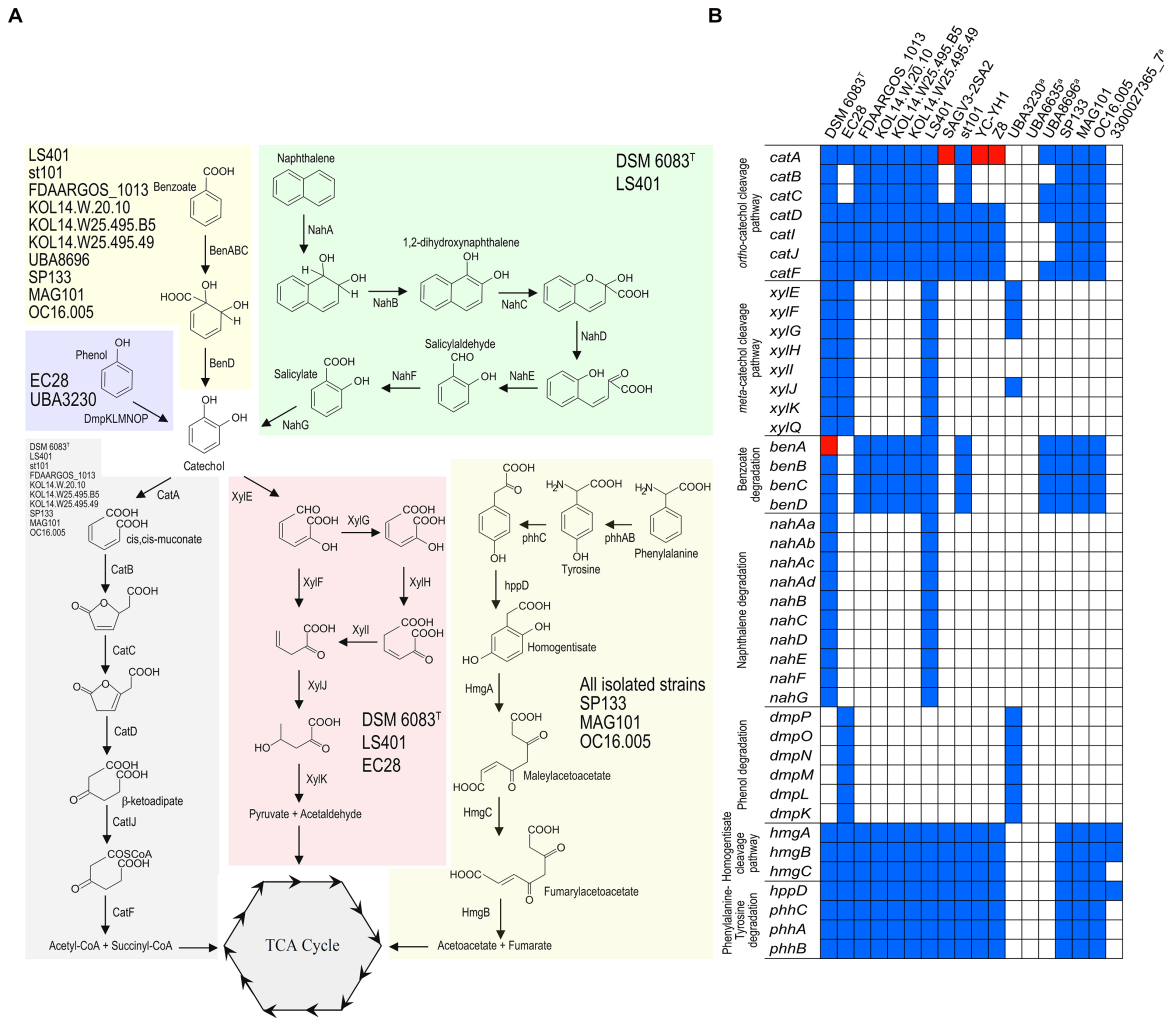
Pathways for degradation of aromatic compounds in *S. balearica*. **(A)** Overview of putative peripheral and ring-cleavage pathways of aromatic compounds found in *S. balearica* genome sequences. Strains containing the full set of genes for a biodegradative route are indicated in each colored box. **(B)** Schematic representation of presence/absence (blue/white coloring) pattern of genes belonging to central and peripheral aromatic pathways in genome sequences of *S. balearica*. Red indicates incomplete or frame-shifted gene. ^a^MAGs with a CheckM completeness estimate lower than 95%.

Seven of the 11 genome sequences from isolated strains and three MAGs codified for the complete catechol *ortho* ring-cleavage pathway (Figure 6), containing the key enzyme catechol 1,2-dioxygenase. This enzyme was described in well-known biodegradative strains, such as *Pseudomonas* sp. strain EST1001 and *Acinetobacter baylyi* strain ADP1, which catabolize a wide range of aromatic compounds (Neidle et al., 1988; Kivisaar et al., 1991). The presence of the catechol *ortho*-cleavage pathway was correlated with benzoate degradation (Figure 6), since the same genomes that contained the complete catechol degradation route harbored genes codifying for a three-component benzoate 1,2-dioxygenase (BenABC) and 1,6-dihydroxycyclohexa-2,4-diene-1-carboxylate dehydrogenase (BenD) (Figure 6; Supplementary Table 4). This is usually required for complete benzoate degradation by bacterial species (Pérez-Pantoja et al., 2012). Exceptionally, the gene encoding the large subunit of benzoate dioxygenase (*benA*) was found to be frame-shifted in the genome sequence of *S. balearica* DSM 6083^T^, suggesting a non-functional benzoate degradation pathway in this bacterium. Of these strains with genes for benzoate degradation, four could be tested for growth on benzoate as a sole carbon and energy source, of which only two (*S. balearica* strain LS401 and strain st101) were able to grow adequately (Table 1). *S. balearica* DSM 6083^T^ did not grow on benzoate, which was expected, due to the frame-shift in the *benA* gene, while *S. balearica* strain CCUG 18844 (= FDAARGOS_1013) grew very weakly, despite harboring all necessary genes and being previously reported as a benzoate degrader (Pichinoty et al., 1977). However, this strain also grew very weakly on phenylalanine, tyrosine (Table 1) and, even, on non-aromatic compounds, such as succinate. Thus, a possible explanation is that the strain may harbor auxotrophic features that result in limited growth on minimal media.

**Table 1:** Growth assays performed on aromatic compounds with the six *S. balearica* strains that are available at the CCUG.

*S. balearica* strain	Benzoate	Phenol	Salicylate	Naphthalene	Phenylalanine	Tyrosine
CCUG 44487^T^ (= DSM 6083^T^ = SP1401^T^)	−	−	+	+	+/−	+
CCUG 66666 (= LS401)	+	−	+	+	+/−	+
CCUG 66667 (= st101)	+	−	−	−	+/−	+
CCUG 70972 (= Z8)	−	−	−	−	+/−	+
CCUG 72049 (= SAGV3-2SA2)	−	−	−	−	+/−	+
CCUG 18844 (= DSM 46326 = FDAARGOS_1013)	+/−	−	−	−	+/−	+/−

−: assays showed no growth after 5 days.

+/−: assays showed very weak growth, in all cases not higher than OD_600_ = 0.1.

+: assays showed distinct growth, in all cases higher than OD_600_ = 0.4.

Three isolated strains (*S. balearica* DSM 6083^T^, LS401, and EC28) encoded for the entire catechol *meta*-cleavage pathway (Figure 6), harboring a key catechol 2,3-dioxygenase similar to those contained in plasmids pWW0, pNAH7 and pDK1. These plasmids are found in *Pseudomonas* species associated with the catabolism of toluene, naphthalene and phenol, respectively (Harayama et al., 1987; Bartilson and Shingler, 1989; Benjamin et al., 1991). Moreover, the genome sequences of two strains (*S. balearica* DSM 6083^T^ and strain LS401), which harbor the catechol *meta*-cleavage pathway, codified all components for channeling naphthalene into catechol (Figure 6), and both strains were able to grow using naphthalene and salicylate as sole carbon and energy sources (Table 1), which is in agreement with a previous study (Rossello et al., 1991). *S. balearica* strain CCUG 66667 (= st101), despite being originally described as naphthalene degrader (Dutta, 2001), was not able to grow on naphthalene as a sole carbon and energy source and no genes for naphthalene degradation were detected in its genome sequence, as previously reported (Salvà-Serra et al., 2017). This suggests that *S. balearica* strain st101 may have lost the naphthalene degradation capacity, which could be related to mobile genetic elements, such as plasmids, transposons, or integrative conjugative elements, being unstable in the absence of selective pressure (Phale et al., 2019). As well, one of the 11 isolate-derived genome sequences (*S. balearica* EC28) and one MAG (*S. balearica* UBA3230) encoded for a multicomponent phenol 2-monooxygenase (Figure 6), comparable to that found in *Pseudomonas* sp. strain CF600, which has been related to phenol catabolism through catechol *meta*-cleavage pathway (Shingler et al., 1989; Nordlund et al., 1990), and that found in *S. chloritidismutans* strain ST-9 (Michael et al., 2017). Additionally, all sequences except various MAGs, harbored the homogentisate ring-cleavage pathway (Figure 6), which is associated with catabolism of the aromatic amino acids tyrosine and phenylalanine (Arias-Barrau et al., 2004). In this context, all six strains were able to grow on tyrosine, but only weakly on phenylalanine as a sole carbon source (Table 1), similar to the phenotype reported in *Pseudomonas putida* strain KT2440, in which growth on phenylalanine as the sole carbon source was almost negligible, in contrast to growth with phenylalanine as a sole nitrogen source (Herrera and Ramos, 2007). Genes encoding for extra 45 peripheral and 18 central enzymes for degradation of aromatic compounds were also searched for but were not detected in the analyzed genome sequences (Supplementary Table 4). However, the pan-genomic analysis indicates that only a fraction of the predicted pool of genes of *S. balearica* has been included in this study and that more strains of *S. balearica* will reveal novel genes and pathways. Additionally, it should be noted that four of the seven MAGs included in this study have low completeness levels (67–68%), which indicates that significant fractions of their genomes were missing. This implies that further pathways and capabilities in *S. balearica* strains for biodegradation of aromatic compounds, complementary to homogentisate- and catechol *ortho-* and *meta*-cleavage pathways, might be revealed in future studies.

### 3.6. Additional genomic features of *Stutzerimonas balearica*

The analysis of the genome sequences of *S. balearica*, using the tool RGI (CARD), did not reveal any antibiotic resistance genes that seemed acquired horizontally (Supplementary Table 4). This could be expected as none of the 18 genome sequences is derived from an environment with apparent high antibiotic selective pressure. However, the natural transformation capacity reported for multiple members of *Stutzerimonas* and related species (Carlson et al., 1983; Lorenz and Sikorski, 2000), could potentially facilitate the acquisition of antibiotic resistance genes when exposed to antibiotic pressure, such as the reported VIM metallo-β-lactamase in a *S. balearica* clinical isolate (Uddin et al., 2022). Indeed, genes codifying for type IV pili components, which are essential for natural genetic transformation (Graupner et al., 2000, 2001), were found in all genome sequences (Supplementary Table 4). The gene *pilAI*, which is located upstream of *pilB*, in opposite direction, and is essential for pili formation (Graupner et al., 2000), was not detected by the software Vfanalyzer. However, a gene encoding a protein with low amino acid identity to PilAI (39% in *S. balearica* DSM 6083^T^) was found upstream of *pilB* in almost all genome sequences. The genes *comA*, also essential for natural transformation, and *exbB*, which inactivation has been shown to decrease the level of natural transformation (Graupner and Wackernagel, 2001), were found also in all isolate-derived genome sequences and five MAGs. This suggests that natural transformation might have played an important role in the evolution and diversification of *S. balearica*. However, future studies are necessary to elucidate the capacity of *S. balearica* for natural transformation.

The RGI analysis revealed subunits of efflux pumps of the resistance-nodulation-cell division (RND) and small multidrug resistance (SMR) families, which can play significant roles in resistance by extruding multiple antibiotics (Bay and Turner, 2016; Murakami, 2016). Meanwhile, the search of antibacterial biocide and metal resistance genes revealed a wide range of genes (Supplementary Table 4), which might be advantageous for strains of *S. balearica* to thrive in diverse polluted and anthropogenic environments. Many were widely distributed among the genomes and MAGs and, indeed, are intrinsic of *Pseudomonas* (e.g., the RND efflux pump MexEF-OprN, which in *P. aeruginosa* has been associated with extrusion of multiple antibiotics but also to organic solvents such as *n*-hexane and *p*-xylene) (Köhler et al., 1997; Li et al., 1998), while others were more strain-specific. For instance, *S. balearica* strain YC-YH1 was the only strain containing (and with high amino acid sequence identity to the reference sequence: greater than 98%) the genes coding for all components of TbtABM, an RND multidrug efflux pump that, in *S. stutzeri*, has been associated with resistance to the tributyltin and decreased susceptibility to various antibiotics (Jude et al., 2004). Meanwhile, *S. balearica* strain EC28 was the only strain carrying the gene *qacE*, which encodes an SMR transporter (Quaternary ammonium compound-resistance protein QacE; also with greater than 98% sequence identity to the reference sequence), which has been associated with resistance to intercalating dyes and quaternary ammonium compounds (Paulsen et al., 1993). A homolog of this protein was also found in the MAG *S. balearica* 3300027365_7, although with lower identity to the reference (73.6%).

Multiple factors that have been associated with virulence in *Pseudomonas* were also detected in *S. balearica* genome sequences (Supplementary Table 4). These included genes related with lipopolysaccharide synthesis, the main antigen (O-antigen) and a major and diverse virulence factor of *P. aeruginosa* (Pier, 2007), which has also been shown to be highly diverse in *Stutzerimonas* (Rosselló et al., 1992). Numerous protein sequences were linked to flagella biosynthesis, in agreement with the description of the species (Bennasar et al., 1996), and which has been linked to biofilm formation in *P. aeruginosa* (O'Toole and Kolter, 1998). Other sequences were related with type IV pili biogenesis, which confers twitching motility and which have also been linked to capacity for biofilm formation in *P. aeruginosa* (O'Toole and Kolter, 1998) and to genetic transformation in *P. stutzeri* (Graupner et al., 2000). The genome sequences also revealed multiple elements related with the regulation and biosynthesis of alginate, which in *P. aeruginosa* has been shown to play an important role in biofilm formation and to protect bacteria from external adversities (Nivens et al., 2001). Genes encoding a complete secretion system were found, homologous to the Xcp secretion system of *P. aeruginosa*, a type II secretion system associated with the secretion of numerous substrates encoded all over the chromosome, including toxins and enzymes (Filloux, 2011). In the case of *S. balearica* DSM 6083^T^, nine of the genes were structured in a single cluster (*xcpR*, *xcpS*, *xcpT*, *xcpU*, *xcpV*, *xcpW*, *xcpX*, *xcpY*, *xcpZ*), while two others were co-located elsewhere in the chromosome (*xcpP*, *xcpQ*). The twelfth gene (*xcpA*), essential for the proper functioning of the Xcp system (Bally et al., 1992), was found in a different region of the chromosome. Additionally, several urease-related genes were detected, including a urea uptake system (*urtA*, *urtB*, *urtC*, *urtD*, *urtE*), urease subunits (*ureA*, *ureB*, *ureC*) and urease accessory proteins (*ureD*, *ureE*, *ureF*, *ureG*, *ureJ*).

The genome sequences also revealed that multiple strains of *S. balearica* contain a CRISPR-Cas system (Supplementary Table 4), which are adaptive immune mechanisms that are present in approximately 10% of bacteria (Burstein et al., 2016). The genome sequence of *S. balearica* DSM 6083^T^ revealed a full CRISPR-Cas system subtype I-E (Makarova et al., 2011), comprising nine CRISPR-associated genes and two CRISPR arrays. One CRISPR array was located upstream of the CRISPR-associated genes and contained 69 direct repeats and 68 non-redundant spacer sequences. The other was located downstream, with six direct repeats and five spacers. No leader sequence was detected for this array, which might be the reason of its low number of spacers (Alkhnbashi et al., 2016; Fernández Juárez, 2017). CRISPR-Cas systems with the same genetic structure and a varying number of spacer/repeats were found in five other genome sequences of *S. balearica* strains and three MAGs. The number of spacer/repeats in the other genome sequences ranged from 16/17 to 103/104. An additional potential CRISPR-Cas system of type IV was found in MAG101, containing a CRISPR array formed by 11 spacers and 12 repeats. Viruses are the most common biological entities in the marine environment and can be significant controlling agents on marine bacterial communities (Fuhrman, 1999). Therefore, CRISPR-Cas systems might be critical systems for certain strains of *S. balearica.* In fact, genome analyses also revealed intact prophages in nine of the 18 genome sequences of *S. balearica*, with sizes ranging from 34 to 83 kb (Supplementary Table 4).

Although no plasmids were revealed in the complete genomes, putative ICEs with type IV secretion systems (T4SS) were predicted in six of the isolate-derived genome sequences and four MAGs. The draft status of most of the genomes impeded the estimation of the size of multiple ICEs that were predicted to be split in multiple contigs or scaffolds. However, those predicted on a single contig or scaffold or in complete genome sequences exhibited sizes ranging from 58 to 269 kb (Supplementary Table 4). Multiple IMEs were also predicted, with sizes ranging from 2.3 to 15.6 kb. In *S. balearica* strain FDAARGOS_1013 (= CCUG 18844 = DSM 46326), three ICEs were predicted, totaling 404 kb (i.e., 9.1% of the genome). In the case of *S. balearica* DSM 6083^T^, the ICE encoded the naphthalene upper pathway and the catechol *meta*-cleavage pathway. This was observed also in the genome of *S. chloritidismutans* AN10 (formerly, *P. stutzeri* genomovar 3) (Bosch et al., 1999, 2000; Hirose et al., 2021). Among others, ICEs and IMEs encode multiple transporter systems, transposase genes, toxin-antitoxin systems as well as numerous hypothetical proteins. In any case, predictions of these mobile genetic elements should be confirmed in future studies.

The genome sequences also revealed that each *S. balearica* strain carries between 306 and 361 regulatory genes (7.7–8.3% of the isolate-derived genome sequences) (Supplementary Table 4). These included tens of two-component systems, transcription factors (i.e., transcriptional regulators, one-component systems, response regulators and sigma factors) and other DNA-binding proteins. These findings are in accordance with previous reports in *Pseudomonas* (Stover et al., 2000) and suggest that *S. balearica* has a large capacity for adapting to varying environmental conditions and responding to external factors.

## 4. Conclusion

*S. balearica* is a diverse species with an open pan-genome that will continue to reveal new genes as the genomes of new strains are sequenced. The detection of 176 strains of the species revealed that *S. balearica* has been found in a wide range of environments, although principally in aquatic and polluted environments, most of which were associated with the oil industry. The genomes of strains of *S. balearica* indicate that the species has a diverse potential for biodegradation of aromatic compounds, which has been experimentally confirmed in six of the strains, and presents multiple features that increase our understanding of the diversity and the lifestyle of *S. balearica*.

## Data availability statement

The strains used in this study are available at the Culture Collection University of Gothenburg (CCUG, Gothenburg, Sweden; www.ccug.se). The draft genome sequence of *S. balearica* strain SAGV3-2SA2 (= CCUG 72049) is deposited and publicly available in DDBJ/ENA/GenBank under the accession no. RAXS00000000. The Illumina sequence reads have been deposited at the Sequence Read Archive (SRA) (Leinonen et al., 2011) under the accession number SRR14574958.

## Author contributions

FS-S, DP-P, JL, and AB-F: conceptualization. FS-S, DP-P, RD, DJ-L, and VF-J: methodology. FS-S, DP-P, RD, DJ-L, and AB-F: validation. FS-S and AB-F: original draft preparation. FS-S, DP-P, RD, DJ-L, VF-J, HE-J, EM, JL, and AB-F: review and editing. HE-J, EM, and AB-F: supervision. EM, JL, and AB-F: project administration. DP-P, EM, JL, and AB-F: funding acquisition. All authors contributed to the article and approved the submitted version.

## Funding

This work was supported by the Spanish MINECO through projects CGL2009-12180 and Consolider CSD2009-00006, as well as funds for competitive research groups from the Government of the Balearic Islands (the last two funds with FEDER co-funding). The DNA sequencing and analytical work was funded, in part, by the CCUG Project: Genomics and Proteomics Research on Bacterial Diversity. The CCUG is supported by the Department of Clinical Microbiology, Sahlgrenska University Hospital and the Sahlgrenska Academy of the University of Gothenburg, Sweden. DP-P and RD acknowledge the support of the FONDECYT 1201741, FONDECYT 11220354, and ANID-PIA/BASAL FB0002 grants of the Chilean government, and the LE19-05 project supported by the Fund of Scientific and Technological Equipment, year 2019, Universidad Tecnológica Metropolitana.

## Conflict of interest

The authors declare that the research was conducted in the absence of any commercial or financial relationships that could be construed as a potential conflict of interest.

## Publisher’s note

All claims expressed in this article are solely those of the authors and do not necessarily represent those of their affiliated organizations, or those of the publisher, the editors and the reviewers. Any product that may be evaluated in this article, or claim that may be made by its manufacturer, is not guaranteed or endorsed by the publisher.

## Supplementary material

SUPPLEMENTARY FIGURE 1 Phylogenetic tree based on partial *rpoD* sequences (549 bp). The tree includes 111 of the 113 genome sequences included in this study; the other two did not contain any *rpoD* sequence in the assembly. The phylogenetic analysis also includes the reference *rpoD* sequences of most of the described species and phylogenomic species of *Stutzerimonas*. The GenBank accession numbers of the genome sequences are listed in Supplementary Table 1 and those of the reference sequences obtained from the PseudoMLSA database are indicated in parenthesis. For each strain, the final taxonomic assignment is indicated. The final phylogenomic species assignment of each strain is indicated in parenthesis. The distances were calculated, using the Jukes-Cantor method, and the tree was constructed, using the neighbor-joining method. Bootstrap values of 50% or greater (from 1,000 replicates) are shown at the nodes. *P. aeruginosa* CCM 1960^T^ was used as an outgroup. Pgs: phylogenomic species; gv.: genomovar; ref.: reference.

SUPPLEMENTARY FIGURE 2 Core genome-based phylogenomic tree of 11 isolate-derived genome sequences and seven MAGs of *S. balearica*, 92 strains of other species and phylogenomic groups of the genus *Stutzerimonas*, and the type strains of *P. indica, P. kuykendallii* and *P. matsuisoli*. The tree was generated, using 35,374 amino acid positions derived from 127 single copy core proteins. Pgs: phylogenomic species; ref.: reference.

SUPPLEMENTARY TABLE 1 List of the 113 genome sequences used in this study and their associated metadata.

SUPPLEMENTARY TABLE 2 ANIb values between the 113 genome sequences (all vs. all) included in this study. Each pairwise comparison was performed bidirectionally; the average value is displayed.

SUPPLEMENTARY TABLE 3 List of the 176 confirmed *S. balearica* strains and their associated metadata.

SUPPLEMENTARY TABLE 4 Genomic features of the genome sequences of *S. balearica*. Sheet S1: Pan-genes of *S. balearica*. Sheet S2: Genes encoding enzymes for catabolism of aromatic compounds detected in one or more genome sequences of *S. balearica*. Sheet S3: Genes for catabolism of aromatic compounds not detected in the genome sequences of *S. balearica*. Sheet S4: Antibiotic resistance genes detected, using Resistance Gene Identifier (RGI) and the Comprehensive Antibiotic Resistance Database (CARD). Sheet S5: Biocide and metal resistance genes predicted by BacMet. Sheet S6: Virulence determinants detected, using VFanalyzer and the VFDB. Sheet S7: CRISPR-Cas systems predicted by CRISPRone. Sheet S8: Prophages predicted by PHASTER. Sheet S9: Integrative and conjugative elements (ICEs) and integrative and mobilizable elements (IMEs) predicted by ICEfinder. Sheet S10: genes associated with natural transformation capacity. Sheet S11: Regulatory genes predicted, using P2RP.

Supplementary File 1 Metagenome assembled genome (MAG) sequence of *S. balearica* UBA3230, annotated using DFAST, in GBK format.

Supplementary File 2 Metagenome assembled genome (MAG) sequence of *S. balearica* UBA6635, annotated using DFAST, in GBK format.

Supplementary File 3 Metagenome assembled genome (MAG) sequence of *S. balearica* 3300027365_7, annotated using DFAST, in GBK format.

## Abbreviations

ANIb, average nucleotide identity based on BLAST
BDBH, bidirectional best-hit
CARD, Comprehensive Antibiotic Resistance Database
CCUG, Culture Collection University of Gothenburg
CDS, coding sequence
COG, cluster of orthologous groups
CRISPR, clustered regularly interspaced short palindromic repeats
dDDH, digital DNA–DNA hybridization
DFAST, DDBJ Fast Annotation and Submission Tool
EC, Enzyme Commission
GGDC, Genome-to-Genome Distance Calculator
GO, gene ontology
GTDB, Genome Taxonomy Database
Gv, genomovar
ICE, integrative and conjugative element
IME, integrative and mobilizable element
MAG, metagenome-assembled genome
NCBI, National Center for Biotechnology Information
MLSA, multilocus sequence analysis
NGS, Next-generation sequencing
OD_600_, optical density at 600 nm
OMCL, Ortho Markov Cluster
PGAP, Prokaryotic Genome Annotation Pipeline
Pgs, phylogenomic species
RGI, Resistance Gene Identifier
RND, resistance-nodulation-cell division
SH-aLRT, Shimodaira-Hasegawa-like approximate likelihood-ratio test
SMR, small multidrug resistance
T4SS, type IV secretion system
VFDB, Virulence Factors Database
