# Supplementary material for: Comparative genomics of Stutzerimonas balearica (Pseudomonas balearica): diversity, habitats, and biodegradation of aromatic compounds
Source: Front Microbiol. 2023 May 15;14:1159176. doi: 10.3389/fmicb.2023.1159176 (PMC10234333; doi:10.3389/fmicb.2023.1159176)
Supplement: Supplementary File 2 — Metagenome assembled genome (MAG) sequence of S. balearica UBA6635, annotated using DFAST, in GBK format. [file Image_1.pdf]

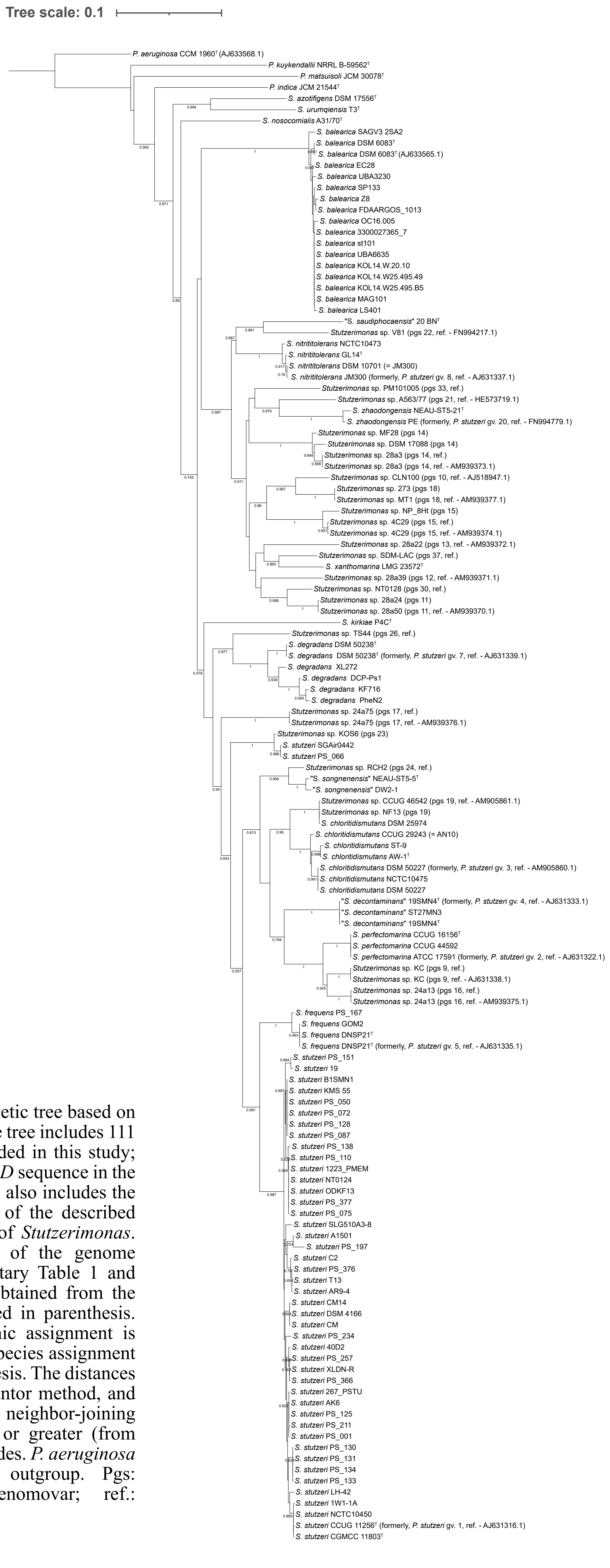

Supplementary Figure 1. Phylogenetic tree based on partial *rpoD* sequences (549 bp). The tree includes 111 of the 113 genome sequences included in this study; the other two did not contain any *rpoD* sequence in the assembly. The phylogenetic analysis also includes the reference *rpoD* sequences of most of the described species and phylogenomic species of *Stutzerimonas*. The GenBank accession numbers of the genome sequences are listed in Supplementary Table 1 and those of the reference sequences obtained from the PseudoMLSA database are indicated in parenthesis. For each strain, the final taxonomic assignment is indicated. The final phylogenomic species assignment of each strain is indicated in parenthesis. The distances were calculated, using the Jukes-Cantor method, and the tree was constructed, using the neighbor-joining method. Bootstrap values of 50% or greater (from 1,000 replicates) are shown at the nodes. *P. aeruginosa* CCM 1960^T was used as an outgroup. Pgs: phylogenomic species; gv.: genomovar; ref.: reference.
