# Supplementary material for: Comparative genomics of Stutzerimonas balearica (Pseudomonas balearica): diversity, habitats, and biodegradation of aromatic compounds
Source: Front Microbiol. 2023 May 15;14:1159176. doi: 10.3389/fmicb.2023.1159176 (PMC10234333; doi:10.3389/fmicb.2023.1159176)
Supplement: Supplementary File 3 — Metagenome assembled genome (MAG) sequence of S. balearica 3300027365_7, annotated using DFAST, in GBK format. [file Image_2.pdf]

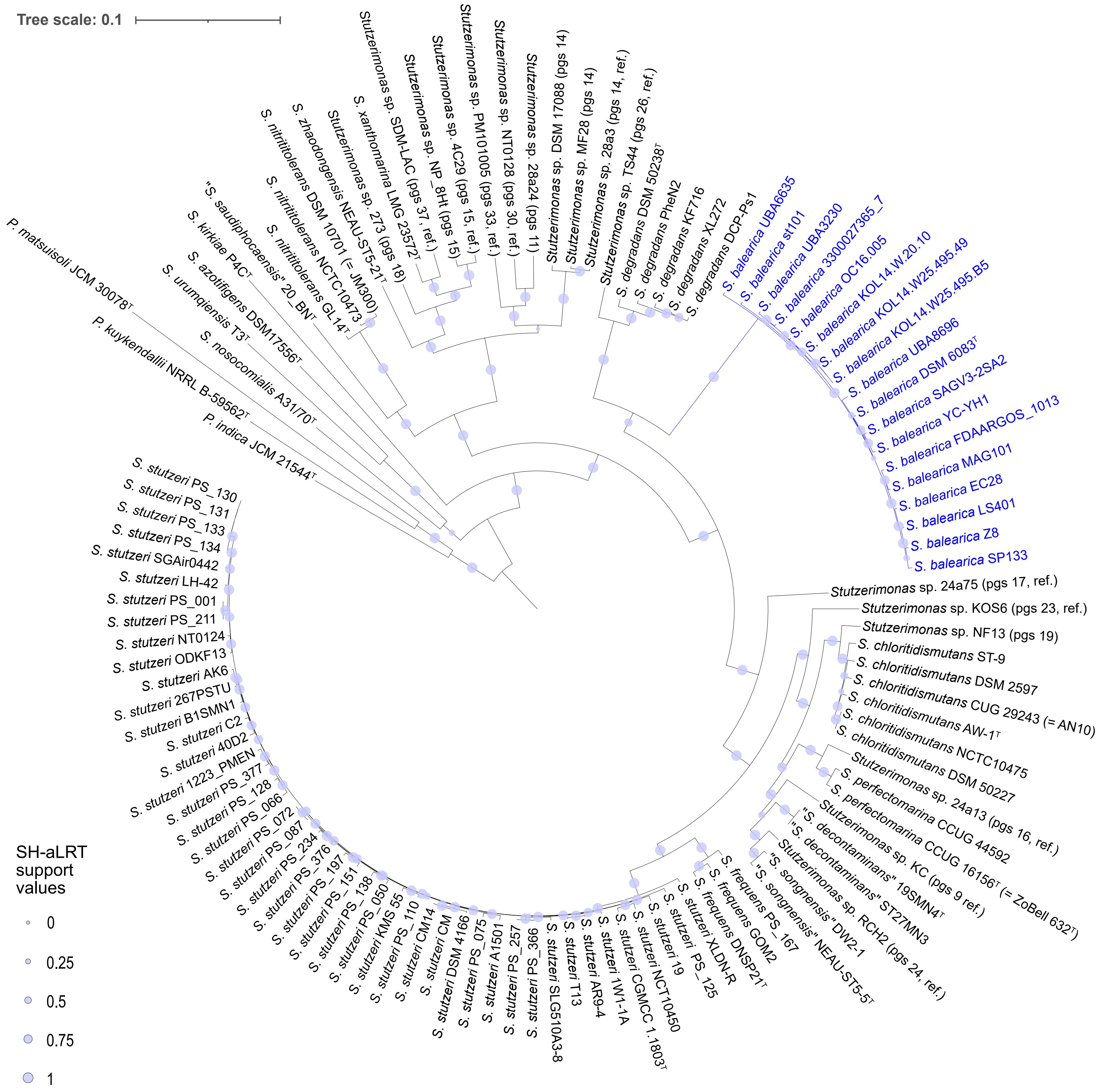

Supplementary Figure 2. Core genome-based phylogenomic tree of 11 isolate-derived genome sequences and seven MAGs of *S. balearica*, 92 strains of other species and phylogenomic groups of the genus *Stutzerimonas*, and the type strains of *P. indica*, *P. kuykendallii* and *P. matsuisoli*. The tree was generated, using 35,374 amino acid positions derived from 127 single copy core proteins. Pgs: phylogenomic species; ref.: reference.
